# Supplementary material for: Positive affect modulates memory by regulating the influence of reward prediction errors
Source: Commun Psychol. 2024 Jun 5;2:52. doi: 10.1038/s44271-024-00106-4 (PMC11332028; doi:10.1038/s44271-024-00106-4)
Supplement: Supplementary file 2 — Supplementary Information [file 44271_2024_106_MOESM2_ESM.pdf]

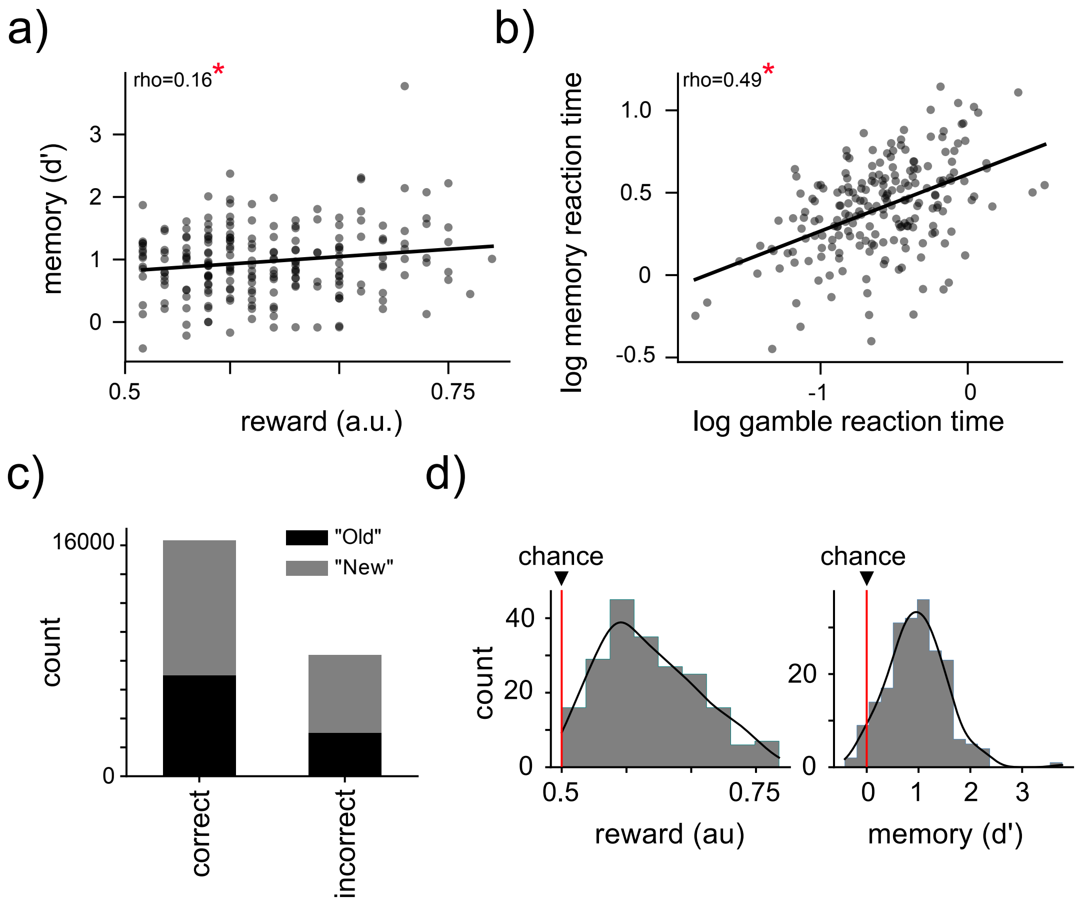

Supplementary Figure 1: **Reaction time and behavioral performance are correlated across tasks.**
a) Correlation of reward in the gambling task with memory performance. Dots denote individual participant values. Line indicates linear model fit to the data. Pearson correlation (rho) also indicated. b) Correlation between gambling and memory reaction times. Dots denote individual participant values. Line indicates linear model fit to the data. Pearson correlation (rho) also indicated. c) Proportion of correct and incorrect memory responses, split by response category. d) Left: histogram of total reward (normalized between 0 and 1) earned by participants, excluding those that performed below chance level, indicated by the red line. Right: histogram of participants' memory performance, excluding those that performed the two-arm bandit task below chance level, measured by d'. Chance-level memory performance is indicated by the red line.

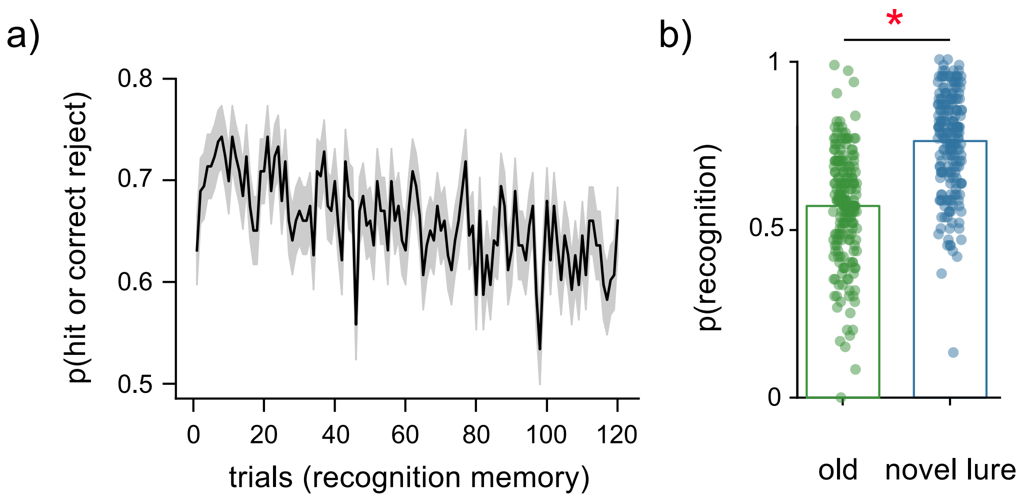

Supplementary Figure 2: **Memory performance across all recognition trials.** a) Overall recognition memory performance, indexed by the probability of either a successful hit or a correct rejection of a novel lure as a function of trial. Shaded lines denote 95% confidence interval. b) Memory performance split by image type (old images vs. novel lure images). For old images, p(recognition) is equivalent to p(hit). For novel lure images, p(recognition) is equivalent to p(correct reject). Novel lures are correctly rejected at a higher proportion than old images are correctly recognized ($z = 31.8$, $p < 0.001$). Asterisk denotes significant difference. Error bars denote standard error. Dots indicate individual subjects.

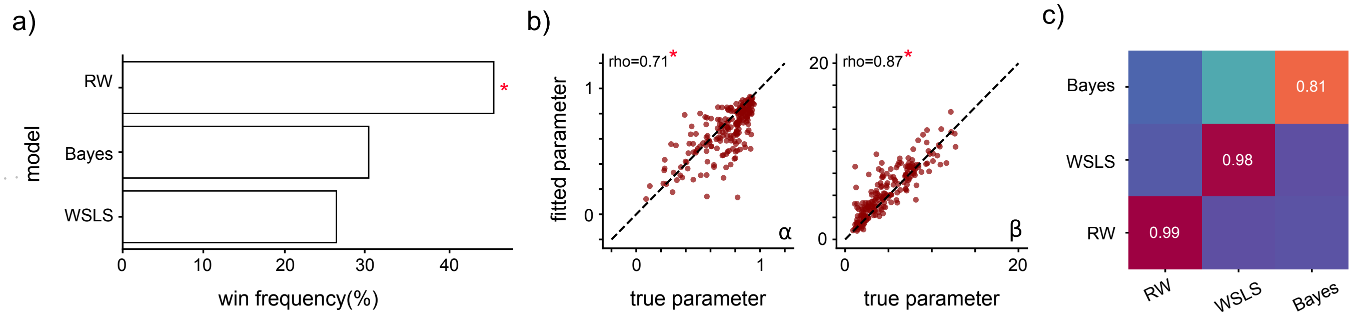

Supplementary Figure 3: **Model selection and validation support RW learning model.** a) Model comparison depicting win frequency for each model when compared using within-subject information criterion (WAIc) for model comparison. Asterisk indicates the most frequent winning model by a significant margin ($\chi^2 = 18.5$, $p < 0.001$, chi-square test of proportions). b) Parameter recovery for the winning model (RW) demonstrating that the parameters for this model are recoverable in the task paradigm. Dotted line denotes diagonal. Pearson correlation (rho) also indicated. Asterisk indicates significance of correlation. c) Confusion matrix depicting the proportion of simulations in which the simulating model was best fit by the same model (diagonal) or alternative models (off-diagonal). The winning model (RW) was recoverable.

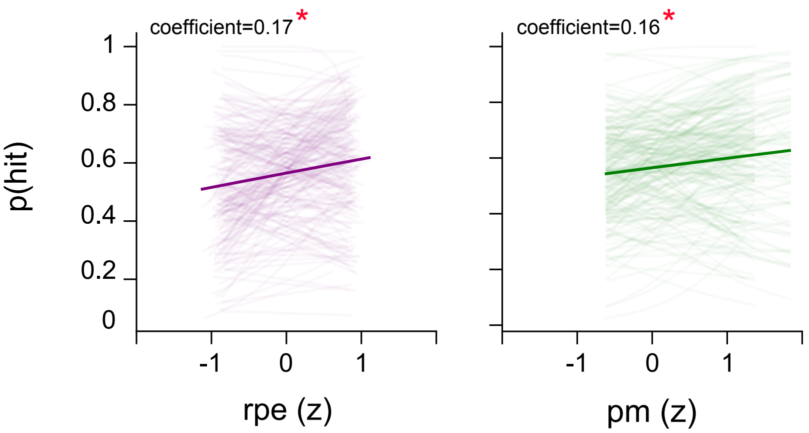

Supplementary Figure 4: **Hit probability as a function of both RPE and PM across subjects.**
Logistic fit of probability of hits as a function of RPE magnitude (left) and PM (right). Dark line indicates model fit to all participants' data. Light lines indicate models fit to individual participants' data. Model coefficients denoted above. Coefficients indicate strength of fixed effects logistic-regression and asterisks indicate significance (both ps < 0.001)

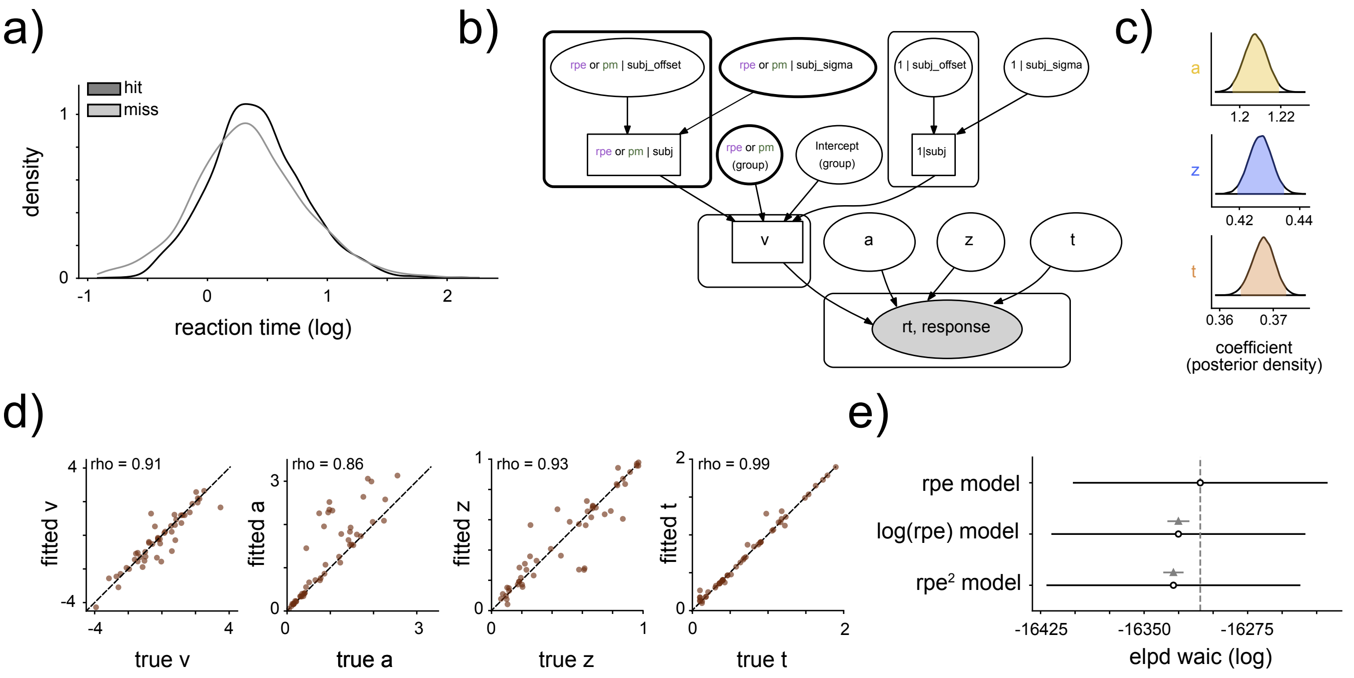

Supplementary Figure 5: **RPE positively modulates drift rate in a drift-diffusion model of recognition memory.** a) Log reaction times for old images during the recognition memory task, split by hits (dark) and misses (light). b) Graphical model of DDM framework. The influence of image features on drift rate (v) were separately tested in individual models that partially pooled the influence of either RPE (purple) or PM (green). c) Model comparison of the two DDMs showing that the model assessing the influence of RPE on drift rate fit the behavioral data better than the model assessing the influence of PM on drift rate. d) Parameter recovery demonstrates good recovery of primary DDM parameters for the RPE-based DDM model.

e) Model comparison of alternative DDM models testing non-linear relationships between drift rate and RPE, showing that the model using a simple linear relationship fits the empirical data best. Gray triangle indicates difference in WAIC scores.

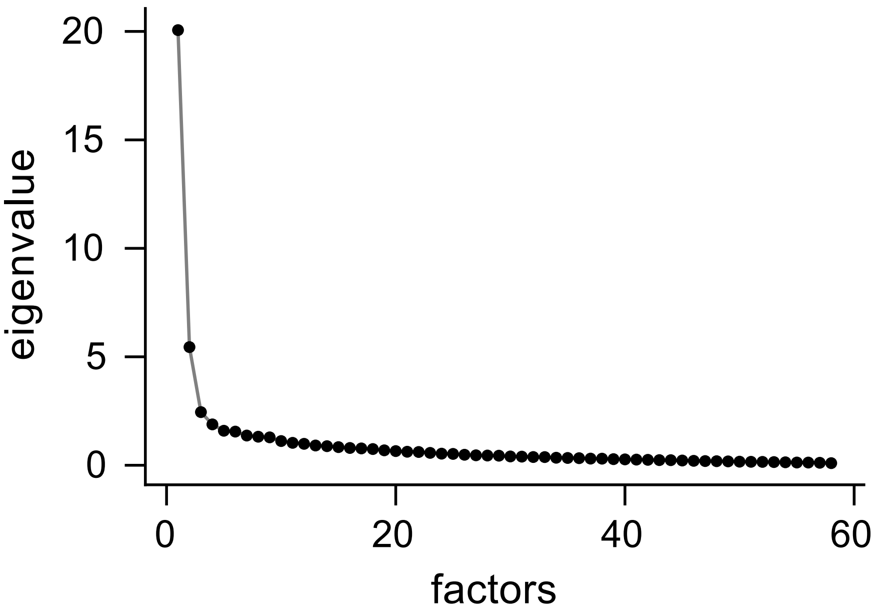

Supplementary Figure 6: **Scree plot for exploratory factor analysis.** Scree plot depicting the eigenvalues for each factor extracted in the factor analysis. Eigenvalues are plotted against factor number, helping to identify the point at which adding more factors provides diminishing returns in explaining variance.

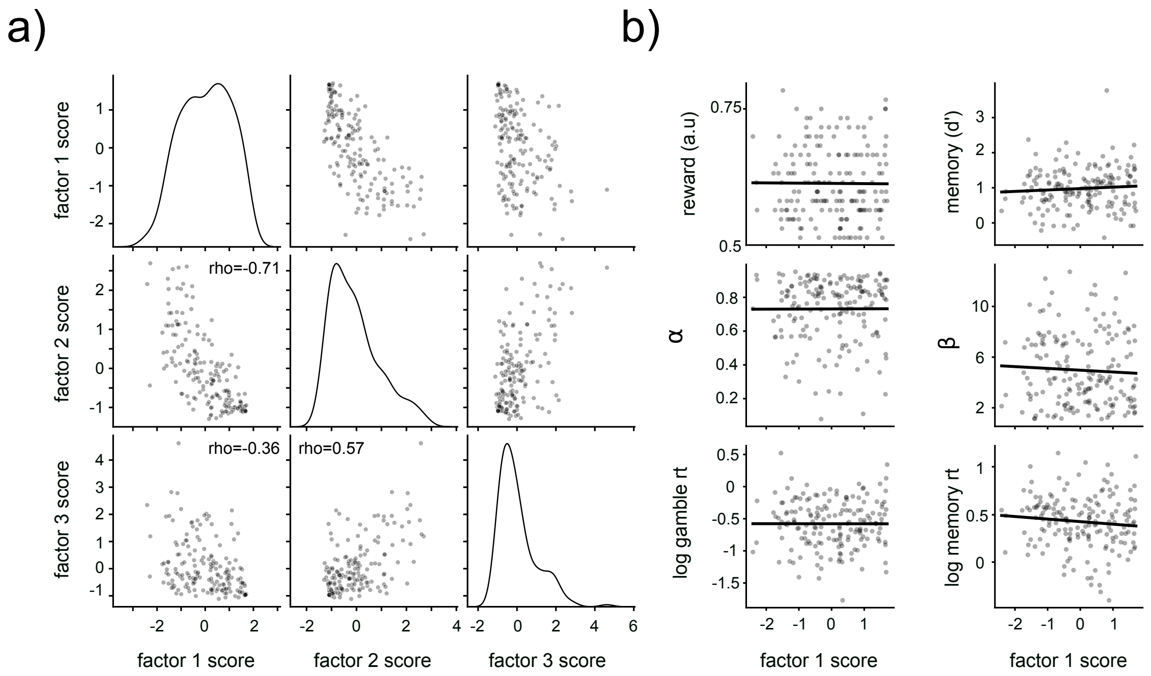

Supplementary Figure 7: **Transdiagnostic mood does not affect subject-level RL traits, task performance, or reaction time.** a) Factor scores across participants. Diagonal: distribution of scores for factor 1 (positive affect), factor 2 (intrusive thoughts and rumination), and factor 3 (obsessive-compulsive behaviors) across participants. Off-diagonal: pairwise scatterplots depicting relationship between scores across scores. b) Correlations between factor 1 score and task performance (top), RL parameters (middle), and reaction times (bottom). Dots denote individual participant values. Line indicates linear model fit.

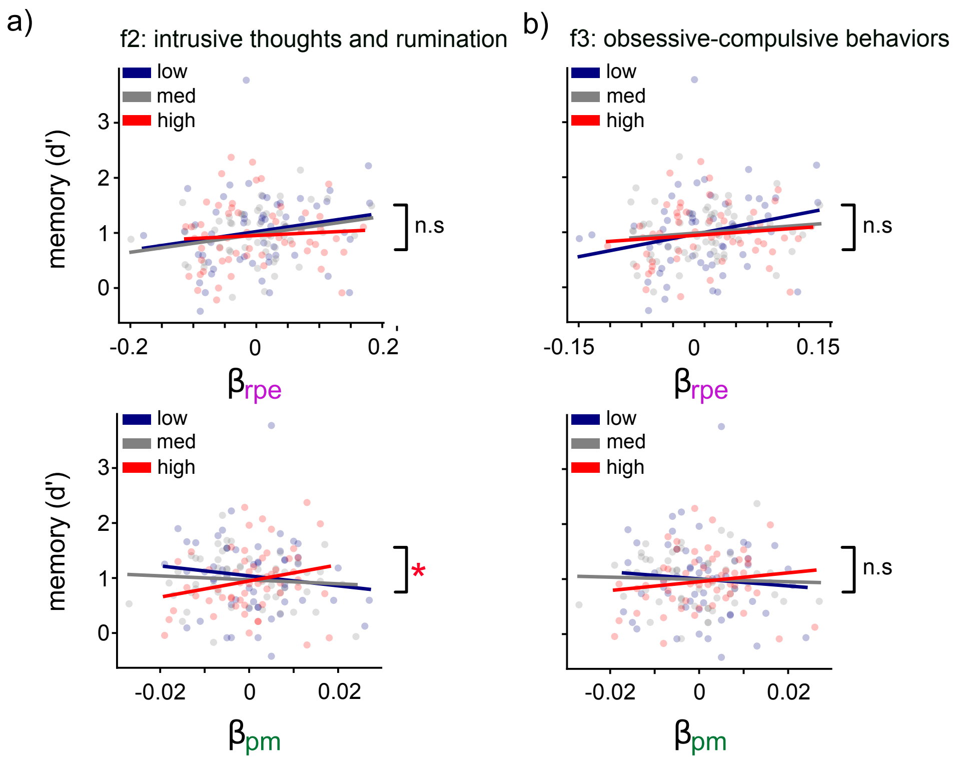

Supplementary Figure 8: **Intrusive thoughts and rumination (f2) and obsessive-compulsive behaviors (f3) do not regulate memory-enhancing effects of RPEs.** a) Top: relationship between β_{RPE} (subject-level random effect from the mixed-effects model) and memory performance, split by a tercile split of affective state (measured by factor 2 score). Bottom: relationship between β_{PM} (subject-level random effect from the mixed-effects model) and memory performance, split by a tercile split of affective state (measured by factor 2 score). Dots denote values for individual participants. Solid line indicates linear model fit to participant data. b) Top: relationship between β_{RPE} (subject-level random effect from the mixed-effects model) and memory performance, split by a tercile split of affective state (measured by factor 3 score). Bottom: relationship between β_{PM} (subject-level random effect from the mixed-effects model) and memory performance, split by a tercile split of affective state (measured by factor 3 score). Dots denote values for individual participants. Solid line indicates linear model fit to participant data.
