# Supplementary material for: Positive affect modulates memory by regulating the influence of reward prediction errors
Source: Commun Psychol. 2024 Jun 5;2:52. doi: 10.1038/s44271-024-00106-4 (PMC11332028; doi:10.1038/s44271-024-00106-4)
Supplement: Supplementary file 3 — Reporting Summary [file 44271_2024_106_MOESM3_ESM.pdf]

nature portfolio

Corresponding author(s): Salman Qasim

Last updated by author(s): May 13, 2024

Reporting Summary

Nature Portfolio wishes to improve the reproducibility of the work that we publish. This form provides structure for consistency and transparency in reporting. For further information on Nature Portfolio policies, see our Editorial Policies and the Editorial Policy Checklist.

Statistics

For all statistical analyses, confirm that the following items are present in the figure legend, table legend, main text, or Methods section.

n/a	Confirmed	
☐	☒	The exact sample size (*n*) for each experimental group/condition, given as a discrete number and unit of measurement
☒	☐	A statement on whether measurements were taken from distinct samples or whether the same sample was measured repeatedly
☐	☒	The statistical test(s) used AND whether they are one- or two-sided<br>*Only common tests should be described solely by name; describe more complex techniques in the Methods section.*
☐	☒	A description of all covariates tested
☐	☒	A description of any assumptions or corrections, such as tests of normality and adjustment for multiple comparisons
☐	☒	A full description of the statistical parameters including central tendency (e.g. means) or other basic estimates (e.g. regression coefficient) AND variation (e.g. standard deviation) or associated estimates of uncertainty (e.g. confidence intervals)
☐	☒	For null hypothesis testing, the test statistic (e.g. *F*, *t*, *r*) with confidence intervals, effect sizes, degrees of freedom and *P* value noted<br>*Give P values as exact values whenever suitable.*
☐	☒	For Bayesian analysis, information on the choice of priors and Markov chain Monte Carlo settings
☐	☒	For hierarchical and complex designs, identification of the appropriate level for tests and full reporting of outcomes
☐	☒	Estimates of effect sizes (e.g. Cohen's *d*, Pearson's *r*), indicating how they were calculated

Our web collection on statistics for biologists contains articles on many of the points above.

Software and code

Policy information about availability of computer code

Data collection	The task was constructed using the publicly available PsychoPy toolbox. Data was collected using the online platform Prolific to be a representative sample of the United States.
Data analysis	Statistical analysis was conducted in Python, using publicly available libraries. Bayesian model-fitting was conducted using pymc, a Python library for Bayesian inference. Drift-diffusion modeling, specifically, was conducted using hssm, a Python library built on top of pymc for constructing sequential sampling models. Bayesian mixed-effects modeling was conducted using bambi, a Python library built on top of pymc for constructing Bayesian regression models.

For manuscripts utilizing custom algorithms or software that are central to the research but not yet described in published literature, software must be made available to editors and reviewers. We strongly encourage code deposition in a community repository (e.g. GitHub). See the Nature Portfolio guidelines for submitting code & software for further information.

Data

Policy information about availability of data

All manuscripts must include a data availability statement. This statement should provide the following information, where applicable:

- Accession codes, unique identifiers, or web links for publicly available datasets
- A description of any restrictions on data availability
- For clinical datasets or third party data, please ensure that the statement adheres to our policy

The behavioral data used in this study are available at: https://osf.io/awu3m/.

Human research participants

Policy information about studies involving human research participants and Sex and Gender in Research.

Reporting on sex and gender	Self-reported biological sex was queried by Prolific for all participants. Sex was included as a control variable in our primary analysis.
Population characteristics	The study sample included 246 online participants (126 female, age=40.1 +- 14.3 years) recruited through the online platform Prolific to be a representative sample of the United States.
Recruitment	Prolific takes the intended sample size and stratifies it across three demographics: age, sex and ethnicity, using census data from the US Census Bureau to divide the sample into subgroups with the same proportions as the national population. Sample size was determined in line with prior research investigating the effects of RPE on memory (Jang et al., Nature Human Behavior 2019).
Ethics oversight	The study was approved by the Institutional Review Board at the Icahn School of Medicine at Mount Sinai.

Note that full information on the approval of the study protocol must also be provided in the manuscript.

Field-specific reporting

Please select the one below that is the best fit for your research. If you are not sure, read the appropriate sections before making your selection.

☐ Life sciences ☒ Behavioural & social sciences ☐ Ecological, evolutionary & environmental sciences

For a reference copy of the document with all sections, see nature.com/documents/nr-reporting-summary-flat.pdf

Behavioural & social sciences study design

All studies must disclose on these points even when the disclosure is negative.

Study description	This study included quantitative behavioral and survey data collected to measure participants' decision-making and recognition memory behaviors as well as psychometric states (depression, anxiety, OCD).
Research sample	The study sample included 246 online participants (126 female, age=40.1 +- 14.3 years) recruited through the online platform Prolific to be a representative sample of the United States.
Sampling strategy	Prolific takes the intended sample size and stratifies it across three demographics: age, sex and ethnicity, using census data from the US Census Bureau to divide the sample into subgroups with the same proportions as the national population. Sample size was determined in line with prior research investigating the effects of RPE on memory (Jang et al., Nature Human Behavior 2019).
Data collection	Data was collected via the online research platform Prolific. Participants used their own computers to perform the task and surveys, and responses were logged by Prolific.
Timing	Data was collected between 01/13/2022-01/14/2022 and 04/07/2022-04/11/2022.
Data exclusions	40 participants were excluded from analysis due to performing at or below chance level, indicating random selections.
Non-participation	None of the participants dropped out or declined participation.
Randomization	Participants were not allocated into separate experimental groups.

Reporting for specific materials, systems and methods

We require information from authors about some types of materials, experimental systems and methods used in many studies. Here, indicate whether each material, system or method listed is relevant to your study. If you are not sure if a list item applies to your research, read the appropriate section before selecting a response.

Materials & experimental systems

n/a	Involved in the study
☒	☐ Antibodies
☒	☐ Eukaryotic cell lines
☒	☐ Palaeontology and archaeology
☒	☐ Animals and other organisms
☒	☐ Clinical data
☒	☐ Dual use research of concern

Methods

n/a	Involved in the study
☒	☐ ChIP-seq
☒	☐ Flow cytometry
☒	☐ MRI-based neuroimaging
